# Comparison of i-Gel as a Conduit for Intubation between under Fiberoptic Guidance and Blind Endotracheal Intubation during Cardiopulmonary Resuscitation: A Randomized Simulation Study

**DOI:** 10.1155/2019/8913093

**Published:** 2019-10-31

**Authors:** Hyun Young Choi, Wonhee Kim, Yong Soo Jang, Gu Hyun Kang, Jae Guk Kim, Hyeongtae Kim

**Affiliations:** Department of Emergency Medicine, College of Medicine, Kangnam Sacred Heart Hospital, Hallym University, Seoul, Republic of Korea

## Abstract

**Purpose:**

This study aimed to compare intubation performances among i-gel blind intubation (IGI), i-gel bronchoscopic intubation (IBRI), and intubation using Macintosh laryngoscope (MCL) applying two kinds of endotracheal tube during chest compressions. We hypothesized that IGI using wire-reinforced silicone (WRS) tube could achieve endotracheal intubation most rapidly and successfully.

**Methods:**

In 23 emergency physicians, a prospective randomized crossover manikin study was conducted to examine the three intubation techniques using two kinds of endotracheal tubes. The primary outcome was the intubation time. The secondary outcome was the cumulative success rate for each intubation technique. A significant difference was considered when identifying *p* < 0.05 between two devices or *p* < 0.017 in post hoc analysis of the comparison among three devices.

**Results:**

The mean intubation time using IGI was shorter (*p* < 0.017) than that of using IBRI and MCL in both endotracheal tubes (17.6 vs. 29.3 vs. 20.2 in conventional polyvinyl chloride (PVC) tube; 14.6 vs. 27.4 vs. 19.9 in WRS tube; sec). There were no significant (*p* < 0.05) differences between PVC and WRS tubes for each intubation technique. The intubation time to reach 100% cumulative success rate was also shorter in IGI (*p* < 0.017) than that in IBRI and MCL in both PVC and WRS tubes.

**Conclusions:**

IGI was an equally successful and faster technique compared with IBRI or MCL regardless of the use of PVC or WRS tube. IGI might be an appropriate technique for emergent intubation by experienced intubators during chest compressions.

## 1. Introduction

Airway management in cardiac arrest is essential for successful resuscitation [1]. Globally, i-gel has been used as a popular supraglottic airway device (SAD) for out-of-hospital cardiopulmonary resuscitation (CPR) [2, 3]. Most SADs except i-gel have a time-consuming process of inflating balloon. However, i-gel does not need to inflate balloon, an advantage which could be beneficial to reduce the time to first ventilation [4]. Recently, i-gel has been widely used, and we frequently encounter out-of-hospital cardiac arrest (OHCA) patients who are already i-gel inserted on arrival to the emergency department.

In CPR for OHCA patients, advanced airway device insertions such as SAD or endotracheal intubation (ETI) are not prior to chest compressions (CCs). When CCs are performed properly, advanced airway device insertion should be considered for optimal oxygenation. Especially in OHCA caused by serious respiratory causes such as acute respiratory distress syndrome or airway obstruction, ETI can be more appropriate than SAD insertion to correct hypoxia and to improve survival ultimately [5].

Nevertheless, ETI using direct laryngoscope during CPR could not be easily achieved even if it is performed by experienced emergency physicians [6]. The vertical motion of glottis caused by CCs interferes in accurate ETI, which can increase hands-off time in CPR [7]. To minimize hands-off time and perform ETI rapidly and accurately, we thought ETI through inserted i-gel could be more advantageous than using direct laryngoscope during CPR.

ETI through inserted i-gel has already been attempted by anesthesiologists in the form of fiberoptic bronchoscope-guided ETI [8–10]. However, it is not an easy technique to handle fiberoptic bronchoscopy for ETI during CPR even for the bronchoscopist. Recently, the novel ETI technique through i-gel without fiberoptic bronchoscope called i-gel blind intubation (IGI) has been introduced and attempted in operative intubation and prehospital resuscitation [11, 12].

We assumed that IGI could minimize interruptions of CCs and achieve successful ETI during CPR. In addition, when using conventional polyvinyl chloride (PVC) endotracheal tube (ETT) in IGI, the PVC tube can be folded and compressed when passing through i-gel. We thought it might result in an increase of hands-off time and decrease of ETI accuracy. So, we assume that the use of wire-reinforced silicone (WRS) tube instead of PVC tube could overcome this problem [13].

This study aimed to compare intubation performances among IGI, i-gel bronchoscopic intubation (IBRI), and intubation using Macintosh laryngoscope (MCL) applying two kinds of ETT during CCs. We hypothesized that IGI using WRS tube could achieve ETI most rapidly and successfully.

## 2. Methods

### 2.1. Study Design

We conducted a randomized crossover manikin study to examine intubation performance using two intubation techniques through i-gel (IGI and IBRI), direct laryngoscopy (MCL), and two ETTs during CCs. This study was performed at Hallym University's simulation center in February 2015. The local ethics committee approved this study in February 2015 (IRB Number: 2015-02-30; the institutional review board (IRB) of Hallym University Kangnam Sacred Heart Hospital). We registered the study protocol in Clinical Trials before study initiation (Clinicaltrials.gov: NCT02411422).

### 2.2. Participants

The sample size was calculated based on a previous study regarding the time required for intubation with CCs [11]. The intubation times (mean ± SD) using i-gel blind intubation was 24.0 ± 9.4 s. To detect a 33% difference in intubation time with a power of 0.9, we estimated that 22 operators would be adequate for each device with a 20% dropout rate. We recruited emergency physicians working at tertiary medical center in February 2015. The inclusion criteria were healthy volunteers (18–60 years) who had more than 50 experiences of intubation using MCL and no experiences for IGI or IBRI. We excluded individuals with wrist or lower back disease. The verbal informed consents were obtained by the participants in this study because the waiver for the written informed consent was approved by the local IRB.

### 2.3. Equipment and Materials

We use direct laryngoscopy (Macintosh blade #4) and i-gel™ (Intersurgical, Workingham, UK, size 4), flexible intubation scope (Ambu® aScope™, Ambu co., Ballerup, Denmark) for ETI with fiberoptic guidance. We use two types of ETT, e.g., PVC tube (Mallinckrodt™ Hi-Lo Oral/Nasal Tracheal Tube Cuffed Murphy Eye, Covidien, Ireland #7.0) and WRS tube (Mallinckrodt™ Oral/Nasal ETT with TaperGuard™ cuff, Reinforced, Covidien, Ireland #7.0).

Participants performed IGI and IBRI using size-4 i-gel (Intersurgical, Workingham, UK) for medium adult (50–90 kg). A flexible intubation scope was used to guide ETT in IBRI. For direct laryngoscope, Macintosh blade (MCL) was used; this device has a size-4 curved blade with a Satin Slip Stylet (Mallinckrodt Medical, St. Louis, MO, USA). Two types of ETTs with an internal diameter of 7.0 mm were used in this study.

We used a high-fidelity manikin (ALS simulator, Laerdal, Stavanger, Norway) to perform CCs and ETI. The normal (nondifficult) airway setting was maintained in the manikin during the study.

The bed-height setting of this study was simulated using a bed (Transport stretcher® No. 747, 76 × 211 cm, 228 kg, Stryker Co., Kalamazoo, MI, USA) with a foam mattress (66 × 192 × 7.6 cm, soft foam with polyurethane covering, Stryker Co., Kalamazoo, MI, USA). A backboard (45 × 60 × 1 cm, 3 kg Lifeline Plastic, Sung Shim Medical Co., Bucheon, Korea) was placed on the bed. The height of the stretcher bed was adjusted to 88.6 cm (bed height: 80 cm + foam mattress: 7.6 cm + backboard: 1 cm) for both ETI and CCs.

### 2.4. Intervention

Instructors gave 1-hour lecture and 2-hour practice for high-quality CPR, ETI using i-gel as blind conduit (IGI), and ETI using i-gel as conduit with fiberoptic guidance (IBRI) for 23 subjects and 4 chest compressors. All instructors were advanced cardiovascular life support (ACLS) instructors certificated by AHA (American Heart Association). They also had more than 500 times experience for ETI and more than 50 times experience for IGI and IBRI. The CPR lecture was constituted with the appropriate chest compression (CC) rate (100–120 bpm), CC depth (5–6 cm), and complete chest recoil and avoiding hyperventilation. The lecture for IGI and IBRI was constituted with the method of i-gel insertion, how to use flexible intubation scope, and the confirmation method of successful intubation. In the IGI and IBRI practice, the subjects were required for more than 10 times drill for IGI and IBRI, respectively. The successful intubation was confirmed by chest rise during bag mask ventilation via ETT. All chest compressors during ETI were AHA-BLS providers. They performed 2 min high-quality CC during ETI on an ALS simulator to prevent fatigue. In the CPR practice, they were requested more than 5 times drill for 2 min hands-only CPR under guidance by ACLS instructors. The CPR quality was monitored by the feedback system of high-fidelity manikin.

After lecture and practice session, subjects were randomly divided into 2 groups by kinds of ETT firstly. For dividing participants to 2 groups, we used drawing lots. The sequence generator (http://www.random.org) was used for random order generation in order to minimize learning effect.

We used two kinds of ETT, one was PVC tube with harder tip and the other was WRS tube with softer tip. Then, subjects rerandomized by a sequence of three ETI methods, i.e., direct laryngoscope, IGI, and IBRI. During ETI, certified basic life support (BLS) provider performed chest compression to the ALS simulator at a rate of 100 to 120 per minute and 5 to 6 cm depth with complete chest recoil. We use airway lubricant (Laerdal, Stavanger, Norway) when i-gel was used as a conduit for smooth insertion.

**Figure 1:**
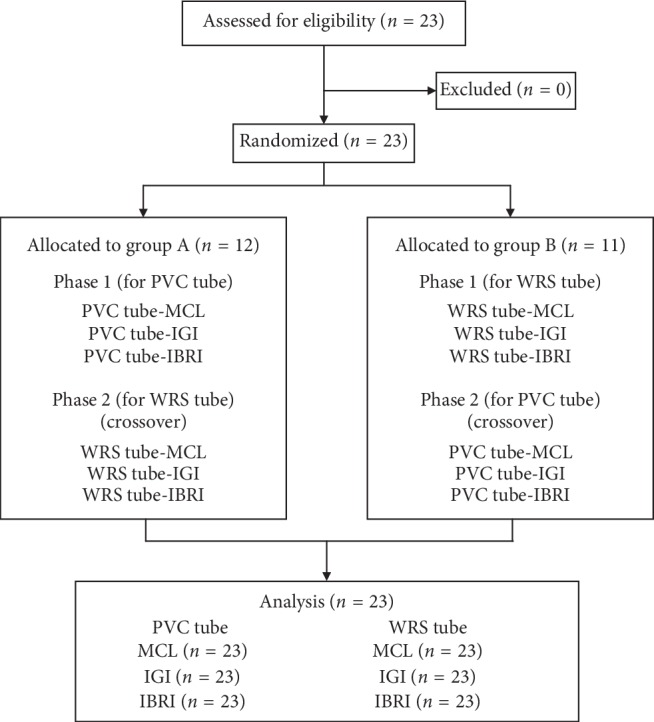
Flow diagram.

Instructor checked and recorded the time from the subject holding a handle of MCL or i-gel to when vocal cord exposed or i-gel was completely inserted (vocal cord exposure time, VET or i-gel insertion time, IIT), when ETT passed the vocal cord (tube pass time, TPT) and when 1^st^ ventilation and chest rising were achieved through ETT by bagging bag-valve mask (1^st^ ventilation time, FVT). (Figure 1).

In the procedure of IGI, subjects inserted i-gel to the ALS simulator, and then ETI was performed using i-gel as a blind conduit. In that order, the subject gave a ventilation through inserted ETT by bagging of bag-valve mask and verified chest rising. Before the subject performed IGI, instructors gave information for successful IGI such as sniffing position and counterclockwise rotation of ETT.

In the procedure of IBRI, we mounted an ETT #7.0 on the bronchoscope before the procedure began. Firstly, subjects inserted i-gel to the ALS simulator, and then subject inserted the bronchoscope to 15 mm connector of i-gel and checked the vocal cord while watching the screen monitor. Then, subjects passed the bronchoscope through vocal cord and verified carina. We pushed the mounted ETT through the bronchoscope and removed it and gave a ventilation through ETT with a bag-valve mask and verified chest rising.

When randomization of three kinds of ETI methods was finished, the subject performed ETI with another type of tube. The sequence of ETI methods was rerandomized.

All recordings were fulfilled by one instructor. We verified successful ETI by inserting the bronchoscope to ETT for visual, confirming carina.

### 2.5. Outcomes

We established intubation time as a primary outcome and cumulative intubation success rate as a secondary outcome.

We regarded esophageal intubation and exceeding 2 minutes from starting intubation to first ventilation as failed intubation. In all ETI attempts, verification of successful ETI using the bronchoscope was done.

### 2.6. Statistical Analysis

The data were collected and arranged using a standard spreadsheet application (Excel, Microsoft, Redmond, WA, USA). Statistical analysis was carried out with the 22.0 version of the Statistical Package for the Social Sciences (SPSS) program for windows (SPSS Inc., Chicago, IL, USA). We described statistics as frequencies and percentages for demographic data and mean ± standard deviation (SD) for continuous data. We used the Shapiro–Wilk test for verifying normal distribution, and we used the Wilcoxon signed-rank test because the result was not according to normal distribution. In addition, we used the Friedman test for comparing three intubation methods and applied Bonferroni's method for post hoc analysis. Kaplan–Meier analysis was performed to analyze the cumulative success rate for intubation time. A significant difference was considered when *p* value was less than 0.05 between two devices and less than 0.017 was considered as significant difference in post hoc analysis of the comparison among three devices.

## 3. Results

Twenty-three subjects participated in this study. The baseline characteristics of the participants are shown in Table 1. ETI failures were recorded only three times in 138 attempts (2.1%), and these failures were all esophageal intubations. There were no cases exceeding 2 min intubation time.

### 3.1. i-Gel Insertion Time (IIT) or Vocal Cord Exposure Time (VET)

IIT in IGI (4.1 ± 1.4 sec) was equal to that in IBRI (4.3 ± 1.2 sec) and significantly shorter than VET in MCL (5.8 ± 2.3 sec) regardless of kinds of ETT (IGI vs. IBRI, *p*=0.277; IGI vs. MCL, *p*=0.001). (Table 2).

### 3.2. Time from Vocal Exposure or i-Gel Insertion to Tube Pass (IIT-TPT)

In the PVC tube, IIT-TPT in IGI (9.8 ± 8.4 sec) was significantly shorter than that of IBRI (20.0 ± 12.2 sec) and longer than that of MCL (7.0 ± 8.9 sec) (IGI vs. IBRI, *p*=0.001; IGI vs. MCL, *p*=0.007). In WRS tube, IIT-TPT in IGI (7.3 ± 2.3 sec) was equal to that in MCL (7.1 ± 4.0 sec) and shorter than that in IBRI (18.8 ± 17.5 sec) (IGI vs. MCL, *p*=0.314; IGI vs. IBRI, *p* < 0.001). There were no differences between PVC and WRS tubes in the comparison of each of the three intubation techniques (MCL, *p*=0.098; IGI, *p*=0.259; IBRI, *p*=0.355). (Table 3).

### 3.3. Time from Tube Pass to First Ventilation (TPT-FVT)

In the PVC tube, TPT-FVT in IGI (3.3 ± 1.0 sec) was significantly shorter than that of IBRI (4.6 ± 0.9 sec) and MCL (7.0 ± 2.0 sec) (IGI vs. MCL, *p* < 0.001; IGI vs. IBRI, *p* < 0.001). Similarly, in WRS tube, TPT-FVT in IGI (3.3 ± 0.9 sec) was also shorter than that of IBRI (4.6 ± 2.0 sec) and MCL (7.0 ± 2.0 sec) (IGI vs. MCL, *p* < 0.001; MCL vs. IBRI, *p* < 0.001; IGI vs. IBRI, *p*=0.001). There were no significant differences between PVC and WRS tubes in the comparison of each of the three intubation techniques (MCL, *p*=0.346; IGI, *p*=0.348; IBRI, *p*=0.291). (Table 3).

### 3.4. First Ventilation Time (FVT, Total Intubation Time)

In PVC tube, FVT in IGI (17.6 ± 8.9 sec) was shorter than that of IRBI (29.3 ± 12.5 sec) and MCL (20.2 ± 9.7 sec) (IGI vs. MCL, *p*=0.016; IGI vs. IBRI, *p* < 0.001). In the WRS tube, FVT in IGI (14.6 ± 3.4 sec) was also shorter than that of IBRI (27.4 ± 19.1 sec) and MCL (19.9 ± 6.7 sec) (IGI vs. MCL, *p* < 0.001; IGI vs. IBRI, *p* < 0.001). There were no significant differences between PVC and WRS tube in the comparison of each of the three intubation techniques (MCL, *p*=0.426; IGI, *p*=0.217; IBRI, *p*=0.189). (Table 3).

### 3.5. Cumulative Success Rate

**Figure 2:**
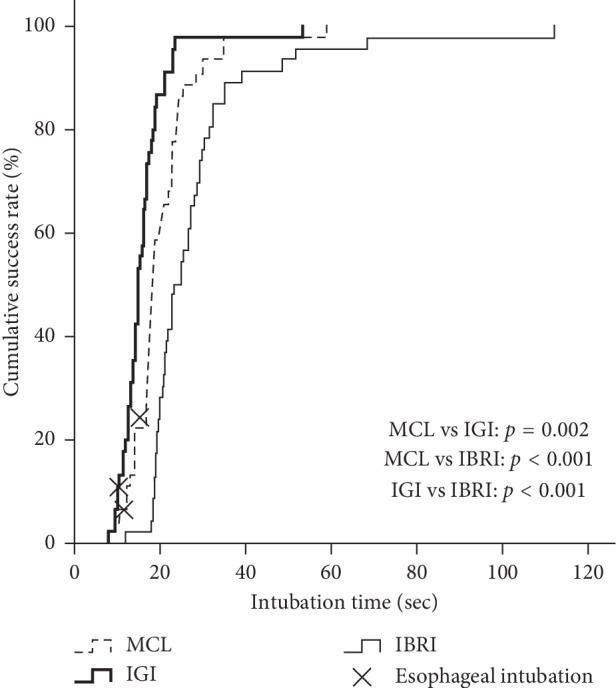
Comparisons of cumulative success rate among three intubation techniques regardless of kinds of endotracheal tubes.

In the comparisons of three intubation techniques, IGI showed significantly shortest time (about 20 seconds) to reach 100% cumulative success rate (IGI vs. MCL, *p*=0.002; IGI vs. IBRI, *p* < 0.001). (Figure 2).

## 4. Discussion

This study demonstrated that the IGI was equally successful and faster technique compared with IBRI or MCL regardless of the use of PVC or WRS tube. To our knowledge, this is the first study which compares the intubation performance of IGI with that of IBRI during chest compressions.

According to the instruction manual for i-gel, IBRI is recommended for i-gel-guided intubation [14, 15]. IBRI may be advantageous to confirm intubation through camera images [16, 17]. However, suggested from the results of this study, to use IBRI, there were some problems. First, IBRI showed longest total intubation time (FVT) among three intubation techniques. It was because the time from i-gel insertion to ETT passing (TPT-FVT) in IBRI was significantly longer than that in IGI and MCL. Second, when attempting IBRI on manikin, participants often did not distinguish trachea from esophagus or they felt some resistance during ETT passing through the internal canal of i-gel. These experiences of participants suggest that intubators can incompletely confirm intubation by IBRI. Furthermore, since IGI and MCL showed equal intubation performance to IBRI, IBRI cannot be first option for emergent intubation during chest compressions.

The blind intubation through SAD such as IGI is not a usual method for emergent intubation in arrest patients [5]. Clinical physicians may select these blind intubations as a rescue technique for intubation failure regardless of the use of MCL or serious intubation conditions by massive blood or vomitus which is unavailable for video laryngoscopes [15, 16]. However, the camera image of IBRI or direct glottic view in MCL can be hindered by blood or vomitus of comatose patients [16]. Thus, IGI can be more appropriate than MCL and IBRI in these serious intubation conditions, because it is not related to camera image or direct vision. Although the intubation time of IGI may be not similar in real world, this study shows the possibility of IGI as a rescue option for arrest patients.

Regarding the nature of blind technique of IGI, the success rate for IGI has been reported from 75% to 100% despite the high speed of IGI [16, 18]. To improve the success rate of IGI, we need to apply counterclockwise rotation technique [11, 19]. This technique improved the success rate of IGI to 100%. Nevertheless, although this technique is applied for IGI in a clinical setting, we expect that the success rate will be lower than that of this study considering anatomical variation or hindrance by blood or vomitus of arrest patients.

In this study, we compared the efficacy of the PVC tube compared with that of the WRS tube in IGI and IBRI. The WRS tube can be more advantageous than the PVC tube, because the WRS tube is more flexible and noncompressible during the passage through i-gel [13, 20]. Nevertheless, the reinforced tube is expensive and less readily available compared with the PVC tube [17, 21]. This study demonstrates that 7 mm sized PVC tube shows similar intubation time compared with 7 mm WRS tube in IGI and IBRI. One previous study also reported that the use of the PVC tube showed lower incidence of postoperative complications including hemodynamic changes or hoarseness compared with those in the WRS tube [20]. Thus, in the clinical setting without the WRS tube, the PVC tube can be attempted for IGI or IBRI by experienced intubators.

This study has some limitations. First, the results of this study were based on manikin simulation. In a clinical setting, there will be several significant factors affecting the intubation performance. These factors include the dynamic hindrance for glottic view during chest compressions, the anatomical variation of airway, and the hindrance by blood or vomitus of arrest patients [16]. Thus, the results of this study may not be guaranteed in clinical situations.

Second, this study is a small sample pilot study for IGI and IBRI. Recently published regarding studies showed similar study design with this study [22–24]. Furthermore, the sample size of this manikin study might be not enough for clinical situation [25]. Therefore, to evaluate accurately the efficacy of IGI, further studies should be performed for humans in the clinical setting with large samples.

Third, although all participants had more than 50 times intubation experiences, the possibility of the experience difference still exists among junior and senior residents and EPs. These unequal experiences for intubation could affect the intubation performance such as intubation time or success rate.

## 5. Conclusion

IGI was an equally successful and faster technique comparing with IBRI or MCL regardless of the use of PVC or WRS tube. Therefore, IGI might be an appropriate technique for emergent intubation by experienced intubators during CCs.

## Figures and Tables

**Table 1:** Baseline characteristics.

	*N* = 23
Age, years	36.2 ± 5.4
Gender, male	19 (82.6%)
Intubators	
EM resident	8 (34.8%)
EP	15 (65.2%)
Experiences	
MCL ≥ 50 times	23 (100%)
i-Gel insertion ≥ 50 times	23 (100%)
Bronchoscopy ≥ 1 times	0 (0%)
IGI ≥ 1 times	0 (0%)
IBRI ≥ 1 times	0 (0%)

EM = emergency medicine; EP = emergency physician; MCL = Macintosh laryngoscopy; IGI = i-gel blind intubation; IBRI = i-gel bronchoscopic intubation.

**Table 2:** Comparisons of intubation time among three intubation techniques regardless of kinds of endotracheal tubes.

	MCL (*n* = 23)	IGI (*n* = 23)	IBRI (*n* = 23)	*p* value^*∗*^	*p* value^§^ IGI vs. MCL	IBRI vs. MCL	IGI vs. IBRI
VET/IIT (sec)	6.1 ± 2.8	4.2 ± 1.3	4.4 ± 1.4	<0.001	<0.001	<0.001	0.12
TPT (sec)	13.2 ± 7.5	12.7 ± 6.4	23.8 ± 15.0	<0.001	0.59	<0.001	<0.001
FVT (sec)	28.0 ± 8.3	16.1 ± 6.7	28.4 ± 16.0	<0.001	<0.001	<0.001	<0.001
VET-TPT (sec)	7.0 ± 6.6	8.5 ± 6.1	19.4 ± 14.9	<0.001	0.008	<0.001	<0.001
TPT-FVT (sec)	6.8 ± 1.9	3.3 ± 1.0	4.6 ± 1.6	<0.001	<0.001	<0.001	<0.001

^*∗*^ *p* < 0.05 considered for significant difference by the Friedman test. ^§^*p* < 0.017 considered for significant difference by the Wilcoxon signed-rank test using Bonferroni's correction. MCL = Macintosh laryngoscopy; IGI = i-gel blind intubation; IBRI = i-gel bronchoscopic intubation; VET = vocal cord exposure time; IIT = i-gel insertion time; TPT = tube pass time; FVT = first ventilation time.

**Table 3:** Comparisons of intubation time among three intubation techniques according to the kinds of endotracheal tubes.

		MCL	IGI	IBRI	*p* value^§^ MCL vs IGI	*p* value^§^ MCL vs IBRI	*p* value^§^ IGI vs IBRI
VET (IIT)	PVC	6.4 ± 3.2	4.3 ± 1.3	4.5 ± 1.6	0.002	0.002	0.205
	WRS	5.8 ± 2.3	4.1 ± 1.4	4.3 ± 1.2	0.001	0.009	0.277
	*p* value^*∗*^	0.615	0.306	0.173			

TPT	PVC	13.5 ± 9.3	14.3 ± 8.3	24.7 ± 12.5	0.543	<0.001	0.001
	WRS	12.9 ± 5.3	11.2 ± 3.2	22.8 ± 17.3	0.173	<0.001	<0.001
	*p* value^*∗*^	0.548	0.114	0.189			

FVT	PVC	20.2 ± 9.7	17.6 ± 8.9	29.3 ± 12.5	0.016	<0.001	0.001
	WRS	19.9 ± 6.7	14.6 ± 3.4	27.4 ± 19.1	<0.001	0.001	<0.001
	*p* value^*∗*^	0.426	0.217	0.189			

VET-TPT	PVC	7.0 ± 8.6	9.8 ± 8.4	20.0 ± 12.2	0.007	<0.001	0.001
	WRS	7.1 ± 4.0	7.3 ± 2.3	18.8 ± 17.5	0.314	<0.001	<0.001
	*p* value^*∗*^	0.098	0.259	0.355			

TPT-FVT	PVC	6.7 ± 1.8	3.3 ± 1.0	4.6 ± 0.9	<0.001	<0.001	<0.001
	WRS	7.0 ± 2.0	3.3 ± 0.9	4.6 ± 2.0	<0.001	0.001	0.001
	*p* value^*∗*^	0.346	0.348	0.291			

^§^ *p* < 0.017 considered for significant difference by using Bonferroni's correction. ^*∗*^*p* < 0.05 considered for significant difference by the Wilcoxon signed-rank test in the comparison between PVC and WRS tubes. MCL = Macintosh laryngoscopy; IGI = i-gel blind intubation; IBRI = i-gel bronchoscopic intubation; VET = vocal cord exposure time; IIT = i-gel insertion time; TPT = tube pass time; FVT = first ventilation time; PVC = polyvinyl chloride endotracheal tube; WRS = wire-reinforced silicone endotracheal tube.

## Data Availability

The data used to support the findings of this study are available from the corresponding author upon request.
